# Proprioceptive Training with Visual Feedback Improves Upper Limb Function in Stroke Patients: A Pilot Study

**DOI:** 10.1155/2022/1588090

**Published:** 2022-01-15

**Authors:** Jieying He, Chong Li, Jiali Lin, Beibei Shu, Bin Ye, Jianhui Wang, Yifang Lin, Jie Jia

**Affiliations:** ^1^Department of Rehabilitation Medicine, Huashan Hospital, Fudan University, Shanghai 200040, China; ^2^National Clinical Research Center for Aging and Medicine, Huashan Hospital, Fudan University, Shanghai 200040, China; ^3^Department of Rehabilitation Medicine, Shanghai Jing'an District Central Hospital, Shanghai 200040, China; ^4^Department of Rehabilitation Medicine, The Shanghai Third Rehabilitation Hospital, Shanghai 200040, China; ^5^Department of Rehabilitation Medicine, Nanshi Hospital Affiliated to Henan University, Nanyang 473000, China

## Abstract

Proprioceptive deficit is one of the common sensory impairments following stroke and has a negative impact on motor performance. However, evidence-based training procedures and cost-efficient training setups for patients with poststroke are still limited. We compared the effects of proprioceptive training versus nonspecific sensory stimulation on upper limb proprioception and motor function rehabilitation. In this multicenter, single-blind, randomized controlled trial, 40 participants with poststroke hemiparesis were enrolled from 3 hospitals in China. Participants were assigned randomly to receive proprioceptive training involving passive and active movements with visual feedback (proprioceptive training group [PG]; *n* = 20) or nonspecific sensory stimulation (control group [CG]; *n* = 20) 20 times in four weeks. Each session lasted 30 minutes. A clinical assessor blinded to group assignment evaluated patients before and after the intervention. The primary outcome was the change in the motor subscale of the Fugl-Meyer assessment for upper extremity (FMA-UE-M). Secondary outcomes were changes in box and block test (BBT), thumb localization test (TLT), the sensory subscale of the Fugl-Meyer assessment for upper extremity (FMA-UE-S), and Barthel Index (BI). The results showed that the mean change scores of FMA-UE were significantly greater in the PG than in the CG (*p* = 0.010 for FMA-UE-M, *p* = 0.033 for FMA-UE-S). The PG group was improved significantly in TLT (*p* = 0.010) and BBT (*p* = 0.027), while there was no significant improvement in TLT (*p* = 0.083) and BBT (*p* = 0.107) for the CG group. The results showed that proprioceptive training was effective in improving proprioception and motor function of the upper extremity in patients with poststroke. This trial is registered in the Chinese Clinical Trial Registry (ChiCTR2000037808).

## 1. Introduction

Proprioception derived from the skin, joints, tendons, and muscle spindle receptors allows us to perceive the movement and position of the body, sense of force, and heaviness [1]. During voluntary movement, the brain integrates the afferent proprioception signals to generate an efficient motor plan and adjust motor performance constantly based on proprioceptive feedback [2]. This process involves the somatosensory and motor systems, which are commonly impaired after stroke. Approximately 50% of patients experience upper limb proprioception deficits after stroke [3, 4], which negatively affect their motor control [5], functional learning [6], daily activity, and participation [7].

Intact proprioception is a critical element to facilitate functional recovery [6]. However, the effectiveness of sensory training for improving upper limb function remains controversial due to the limited studies and heterogeneity of interventions and measures [8–11]. There are limited studies focused on upper limb proprioceptive training in stroke patients compared with the number of studies focused on motor task interventions. Several studies have proposed active multisensory retraining programs that focus on intensive and repetitive sensory tasks with feedback to improve sensory impairment after stroke [12–15]. These training methods generally involve proprioception and tactile discrimination [10]. Furthermore, recent studies have started to investigate the effectiveness of pure proprioception training on patients' outcomes with poststroke [16–18]. Öcal et al. [16] found improved upper extremity motor function in stroke patients after six weeks of proprioceptive training, including the rhythmic stabilization method and active joint position sense training. Vahdat et al. [17] designed robot training techniques with verbal feedback to train the joint position sense of patients with poststroke and demonstrated that it could induce functional connectivity changes in sensorimotor networks. While these studies have shown promising results, Chanubol et al. [19] compared the effectiveness of Perfetti's cognitive sensory motor training therapy, which mainly focuses on active joint position and movement training and tactile training, to conventional treatment. The result showed both groups of patients had significant improvement in hand and arm function, but no significant differences were found between groups.

The conflicting results from previous studies indicate the need for developing a more well-designed approach of proprioception training. Systematic reviews recommend that proprioceptive training involving passive and active movements with or without visual feedback might be more beneficial for improving sensorimotor function [20]. Currently, most proprioceptive interventions focused on active position sense and movement discrimination tasks, but rarely involved passive movement. In addition, most sensory trainings focused on increasing sensory input in the affected side, ignoring or restricting the use of the unaffected side [12, 17]. Recent studies indicate bimanual movements training improves the motor impairments of the affected upper limbs in stroke patients by facilitating cortical and neurologic plasticity [21, 22]. Bimanual movements training is a treatment approach that involves performing repeating tasks with both upper limbs of patients. The training protocol in our study combined passive movement with bimanual movements training. Therapists mobilized both hands of patients in a mirror-symmetrical pattern. Hypothetically, simultaneous passive movement of patients' hands might activate bilateral sensory and motor areas, which improve the sensory performance on the affected limb [23, 24].

Another limitation of previous studies is that they usually used eye masks or verbal instructions to control patients' vision, which was inconvenient for both researchers and patients and affected the efficiency of training [12, 16, 19]. The use of vision is essential in improving the function of the affected hand during training, which includes providing feedback and observing actions. Highly repetitive sensory tasks accompanied by visual feedback in every performance could enhance the training effects [25, 26]. In addition, observing movement or stimulation could modulate the activation of the sensory cortex [27, 28]. Further study is needed to optimize the visual feedback in upper limb proprioceptive training for clinical practice.

In the present study, we developed a proprioceptive training that includes passive and active movements and designed a visual-based sensory training setup to provide manipulable visual feedback. This setup enriches traditional sensory training by enhancing the fundamental role of repetitive visual feedback on sensory tasks. The study is aimed at investigating the feasibility and effectiveness of the proposed proprioception training on upper limb function in patients with poststroke.

## 2. Materials and Methods

### 2.1. Visual-Based Sensory Training Setup

**Figure 1:**
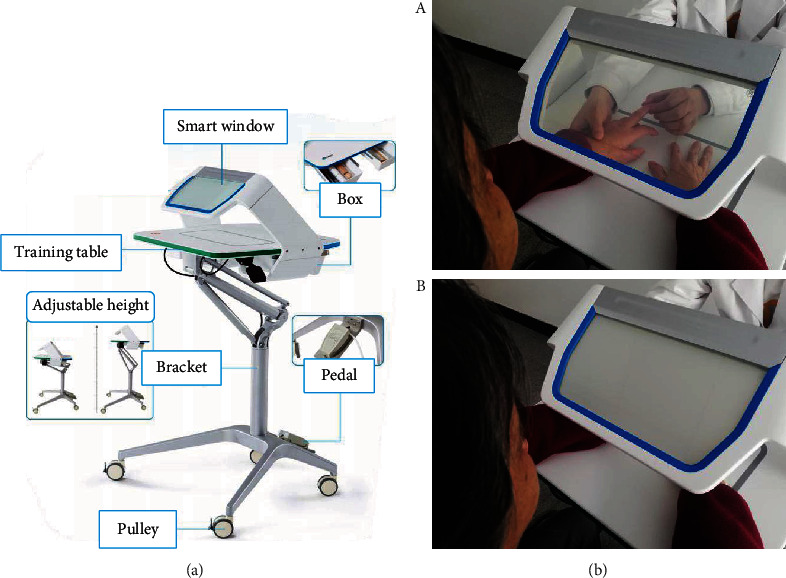
The visual-based sensory training setup. (a) The appearance and composition of the setup. (b) The smart window is in the transparent mode (A) and the opaque mode (B).

The visual-based sensory training setup (700 mm × 800 mm × 1050 mm), also referred to as the perception window, used a smart window to provide manipulable visual feedback (Figure 1(a)). The smart window was fixed on the top of the setup at an angle of approximately 45° with the patient's hands placed naturally under the smart window. The smart window uses electrochromatic technology which allows changing between transparent and opaques modes by applying voltage. A footswitch, mounted under the setup, was used to switch between modes. When therapists depressed the pedal, the power was on and the smart window was transparent. Patients could observe their hands clearly through the window. When therapists removed their feet from the pedal, the power was cut off and the smart window was in opaque mode. At this point, the vision of patients was blocked by the window. During proprioception training, patients did not have to close their eyes but instead watched the smart window in front of them. Therapists conducted proprioceptive training with their hands on the table while modulating the smart window with their feet to control the patient's vision. Instead of giving verbal instructions (“eyes open” and “eyes closed”) or using an eye mask, this setup made it easier to present visual feedback and facilitated patients in achieving a better sense of engagement.

### 2.2. Participants

Participants were recruited from three inpatient rehabilitation departments: Shanghai Jing'an District Central Hospital, Nanshi Hospital Affiliated to Henan University, and Shanghai Third Rehabilitation Hospital. The inclusion criteria were as follows: (1) first-time stroke at least three months ago, (2) age between 18 and 85 years, and (3) unilateral hemiparesis. Patients were excluded if they had one of the following: (1) history of other neurologic diseases and (2) severe visual impairments or aphasia per National Institute of Health Stroke Scale (NIHSS). All patients understood the procedure of the study and signed informed consent forms, which were approved by the ethical committee of Shanghai Jing'an District Central Hospital.

### 2.3. Sample Size

The sample size was calculated by GPower V.3.1.9 (University of Kiel, Kiel, Germany) software. Based on a previous study, a sample size of 18 patients per group was required to achieve 5% alpha and 90% power with the standardized effect size of 1.142 [12]. Considering the possible loss rate of 20%, 20 patients per group were needed to enroll in this study.

### 2.4. Experimental Design

**Figure 2:**
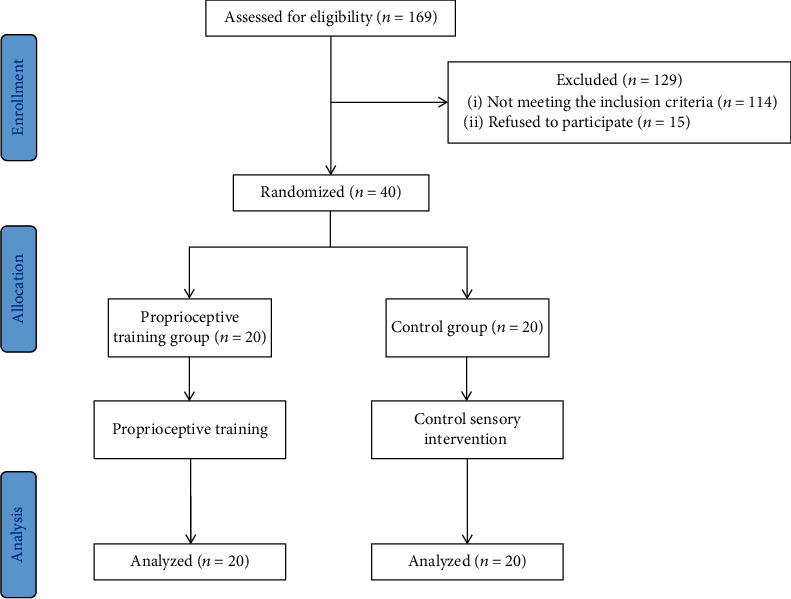
Flow chart of the participant selection and assignment in this study.

A multicenter, single-blinded, prospective parallel-group controlled trial was completed in this study. Before the start of recruitment, treating therapists received uniform training to ensure the consistency of intervention. An independent therapist blinded to the allocation conducted baseline and outcome assessments. All patients were stratified based on the motor subscale of the Fugl-Meyer assessment for upper extremity (FMA-UE-M) (cutoff score was 28) and sex. According to the computer-generated randomization sequence, participants were randomly assigned to the proprioceptive training group (PG) or the control group (CG). The researcher performed randomization by giving treating therapists a sealed opaque envelope when the participant was enrolled. Patients did not contact the allocation researcher, which ensured that the assessment therapist was blinded to the allocation sequence (Figure 2).

The patients assigned to PG received proprioceptive training conducted with the perception window. Before proprioceptive training, the therapist performed stretching and pressure at joints in the upper limb to help patients relax and reduce muscle tone. Both hands of patients were placed on the table. The therapist sat opposite to patients and adjusted the height of the table to ensure that patients could see their hands, wrist, and part of the forearm when the window was transparent. Depending on the patient's somatosensory function status, the therapist tailored individual training with the perception window. In the beginning, the therapist conducted flexion-extension in both upper limbs of patients with the same rhythm and instructed patients to observe and focus on the sensory input on both hands. When the patients reported their perception of the movement in both upper limbs, the therapist switched the smart window to opaque mode, still performing the same movements, and instructed patients to imagine that feeling. If patients could reliably perceive the movement of affected limbs, they were instructed to discriminate the direction of movement in fingers or wrist. Then, patients were instructed to answer which finger was moved or touched by the therapist with their vision occluded. During the training, patients were requested to relax and focus on the sensation that therapists applied with vision occluded. After each movement, the therapist asked patients what they felt and gave them visual feedback by switching the smart window into transparent mode. Based on the performance of the patient, the therapist was able to increase the difficulty of training stage-by-stage, i.e., decreasing the range of limb movement or moving two fingers simultaneously. In the last stage of training, therapists moved the unaffected limb of patients to different positions and requested patients to imitate the position of the unaffected limb with the affected limb. If patients could not match the position, therapists switched the smart window into transparent mode and repeated the training. At the end of the proprioceptive training, the therapist conducted some stretching and pressure in the upper limb again. Every patient in the PG received a 30-minute proprioceptive training per day, five days a week, for four weeks.

Patients allocated to the CG received a 30-minute control sensory intervention (5 times per week for four weeks) provided by experienced therapists. During the sensory control intervention, the smart window was maintained in transparent mode and patients could see their arms under the window. The therapist conducted some pressure and passive movement in the upper limb of patients. In addition, patients were exposed to nonspecific sensory stimulation by grasping objects of different shapes, textures, weights, and sizes. During training, the therapist did not provide verbal instruction, and patients could open or close their eyes according to their wishes. Exposure was taken as a control intervention in a previous study since it was similar to activities of daily living and would not provide extra benefit in most patients with poststroke.

Both groups of patients underwent a 4-hour rehabilitation program (5 times per week for four weeks) provided by experienced therapists. This rehabilitation involved conventional physical and occupational therapy as deemed necessary to address the functional impairment of patients.

### 2.5. Outcome Measures

Patients were assessed at baseline and after four weeks of intervention. The primary outcome was the change in the motor subscale of the Fugl-Meyer assessment for upper extremity (FMA-UE-M), which is a highly reliable and valid assessment that measures motor recovery of the upper limb after stroke [29, 30]. It consists of 33 items, which can be divided into 4 subsections: shoulder-arm, wrist, hand, and upper limb coordination. Each item is scored by a 3-point ordinal scale (0, absent; 1, partial impairment; 2, no impairment). The total score range is from 0 to 66 points [31]. A higher score indicates a better functional performance. The minimal clinically important differences (MCID) of FMA-UE is 9 points in patients with poststroke [32].

Secondary outcomes included changes in the thumb localization test (TLT), the sensory subscale of the Fugl-Meyer assessment for upper extremity (FMA-UE-S), box and block test (BBT), and Barthel Index (BI). TLT was used to assess proprioception. Patients closed their eyes, and the examiner moved the affected upper limb of patients to three different positions and asked patients to pinch the thumb with the unaffected hand. Scoring for each test was based on the patient's performance (0, pinch the thumb accurately; 1, miss the thumb by several centimeters and immediately correct; 2, find the arm and pinch the thumb by tracing from arm; 3, unable to find the thumb). The worst performance of the three tests was the final score [33, 34]. The assessment has established high interrater reliability [35]. FMA-UE-S is scored on a 3-point ordinal scale (0-2) scored to measure light touch and proprioception on the upper extremity [31]. The light touch was tested on the patient's arms and the palmar surface of the hands. The proprioception was tested on the shoulder, elbow, wrist, and thumb of patients. The patient was blindfolded and asked whether they felt the light touch or the position of joints. The total score of FMA-UE-S is 12 [36, 37]. BBT was used to measure gross manual hand dexterity, which is a highly reliable test [38]. The test involves 150 colored cubes and one wooden box divided by a partition into two separate compartments. Patients were instructed to grasp the cube from one compartment and release it into the opposite compartment one by one. The number of cubes patients transferred with their affected hands in one minute was recorded as the score [39]. The performance of basic activities in daily living was assessed with the Barthel Index (BI) [40], which is a reliable measurement [41]. The scale consists of 10 activity items, and the score for each item is based on the patient's degree of independence. Score ranges from 0 (total dependence) points to 100 points (total independence).

The experience of the patient was assessed with the meCUE (modular evaluation of key Components of User Experience, http://www.mecue.de), which has been used in several studies [42, 43]. The questionnaire has four validated modules: product perceptions, user emotions, consequences of use, and overall evaluation [44]. In the modules of product perceptions, user emotions, and consequences of use, each item has three concerning statements evaluated by a 7-point Likert scale with scores ranging from 1 (strongly disagree) to 7 (strongly agree). Considering the characteristics of the perception window, items on “commitment, status, product loyalty” in the original scale were removed in this study. In the module of the overall evaluation, the scale ranges from −5 (bad) to 5 (good), which evaluated the overall experience of the product.

### 2.6. Statistical Analyses

Statistical analyses were processed using SPSS version 26 (IBM Inc., Chicago, IL, USA). We analyzed the normality of the patients' demographic and clinical characteristics with the Shapiro-Wilk test. Data are presented as the mean ± standard deviation (SD) for normally distributed continuous variables. Nonnormally distributed variables or ranked variables are expressed as medians (interquartile ranges, IQRs). The chi-square test was used to compare sex, side of the lesion, and type of stroke between the two groups. The independent Student *t*-test was used to compare age between the two groups, and the independent Mann–Whitney *U* test was used to compare time from stroke and Brunnstrom stages for upper limb and hand. The paired Student *t*-test and Wilcoxon signed-rank test were used for analyzing the pretest and posttest scores in clinical outcomes of both groups. To compare the effect of PG with CG, the changes in continuous outcomes between groups were analyzed by Mann–Whitney *U* test. For the modified meCUE, the results of each subscale were calculated by the mean scores of patients. A *p* value of 0.05 was set for the level of statistical significance.

## 3. Results

From October 2020 through May 2021, forty patients who met the inclusion criteria were enrolled. The flow chart of the participant selection and assignment is shown in Figure 2. Patients were randomly allocated to the PG (*N* = 20) and CG (*N* = 20). There were no adverse events during the intervention. The demographic characteristics of participates are shown in Table 1. The mean age was 59.1 years, and the median time from stroke was 21 weeks. There were no differences in age, sex, side of stroke, time poststroke, stroke type, Brunnstrom stage, or somatosensory performance between groups at baseline.

### 3.1. Effects of Intervention

**Figure 3:**
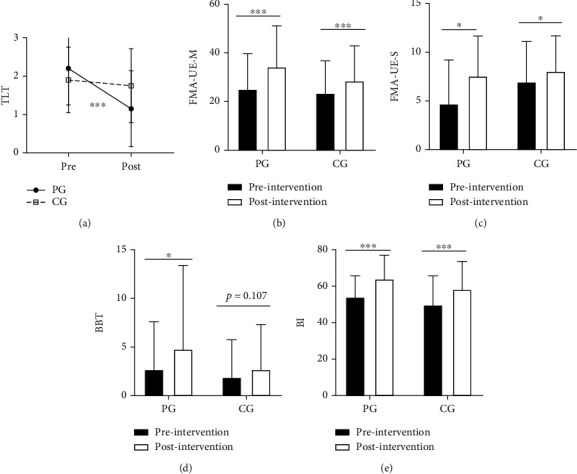
Group differences in clinical measurements. (a) The scores of the thumb localizing test (TLT) in the two groups before (pre) and after (post) intervention. (b) The scores of the motor subscale of Fugl-Meyer assessment for upper extremity (FMA-UE-M) in the two groups before (pre) and after (post) intervention. (c) The scores of the sensory subscale of Fugl-Meyer assessment for upper extremity (FMA-UE-S) in the two groups before (pre) and after (post) intervention. (d) The scores of box and block test (BBT) in the two groups before (pre) and after (post) intervention. (e) The scores of Barthel Index (BI) in the two groups before (pre) and after (post) intervention (^∗^*p* < 0.05 and ^∗∗∗^*p* < 0.001).

In the PG group, patients showed a significant improvement in FMA-UE-M (*p* < 0.001), FMA-UE-S (*p* = 0.001), BBT (*p* = 0.027), TLT (*p* < 0.001), and BI (*p* < 0.001). In the CG group, patients showed a significant improvement in FMA-UE-M (*p* < 0.001), FMA-UE-S (*p* = 0.006), and BI (*p* < 0.001), but there was no significant improvement in TLT (*p* = 0.083) and BBT (*p* = 0.107). These results are shown in Figure 3.

**Figure 4:**
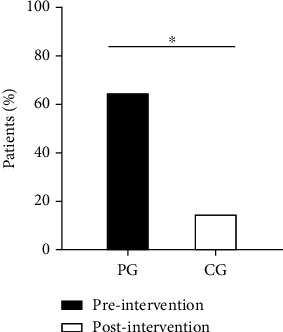
Comparison of the percentages of patients who achieved the minimal clinically important differences (MCID) of the FMA-UE-M between groups after intervention.

The average FMA-UE-M scores improved from 25.0 ± 14.6 to 34.3 ± 16.8 in the PG group and from 23.5 ± 13.3 to 28.5 ± 14.4 in the CG group. In comparison, the mean score changes of FMA-UE-M improved more in the PG than in the CG group (*p* = 0.010), and the percentages of patients who achieved the MCID of the FMA-UE-M were higher in the PG than in the CG group (*p* = 0.001) (Figure 4). The average TLT scores improved from 2.3 ± 0.7 to 1.2 ± 1.0 in the PG group but only from 2.0 ± 0.6 to 1.9 ± 0.8 in the CG group. The average of the FMA-UE-S improved from 4.7 ± 4.5 to 7.6 ± 4.1 in the PG group and from 7.0 ± 13.3 to 8.1 ± 3.6 in the CG group. Compared to the CG group, the PG group showed more improvement in the mean score changes of the TLT (*p* < 0.001) and the FMA-UE-S (*p* = 0.033). Besides, in the PG group, the average BBT scores improved from 2.7 ± 4.9 to 4.8 ± 8.6 and the average BI scores improved from 54.0 ± 11.7 to 65.8 ± 13.9. In the CG group, the average BBT scores improved from 1.9 ± 3.9 to 2.7 ± 4.6, and the average BI scores improved from 50.3 ± 15.5 to 58.3 ± 15.4. We found no significant differences in the mean score changes of BBT (*p* = 0.640) and BI (*p* = 0.134) between the two groups.

### 3.2. Patient Experience

After the last treatment session, patients in the PG completed the modified meCUE to express their experience. The rates of usefulness, usability, and visual aesthetics in product perception were high. For emotion, positive emotion was rated higher than negative emotion. None of the patients felt uncomfortable or pain during the training. Most of the patients indicated that they were willing to carry out the treatment with the setup. The median rate for overall evaluation rated was “3”, which suggested that most patients were satisfied with the treatment (Table 2).

## 4. Discussion

Our results showed that proprioceptive training is effective in improving upper extremity sensorimotor function in patients with poststroke. In addition, most patients in the PG were satisfied with the proposed visual-based sensory training setup. We suggest that proprioceptive training including both passive and active movements with a visual-based sensory training setup is a feasible treatment and could be an adjunct therapy in clinical intervention.

The PG showed a significant improvement in proprioception following training compared with the CG. This finding is in line with results from other studies [12, 13]. It is well known that sensory stimuli are involved in preparing motor plans and monitoring motor performance [2]. Somatosensory deficits impact the motor output of the patient [5–7]. Therefore, the patient needs to receive continuous and systematic proprioceptive training. In this study, FMA-UE-M and BBT data demonstrated that proprioception training was effective in recovering upper limb motor function after stroke. Emerging evidence indicates that proprioceptive training could improve somatosensory and motor function after stroke [20, 45]. Vahdat et al. [17] observed that even a single session of proprioceptive training could modulate functional connectivity in the sensorimotor network, which might be one of the mechanisms for which proprioceptive training improves motor function following stroke. However, there was no significant difference in the improvement of BI between the two groups. The Barthel Index measures the functional ability of a patient in performing daily activities, which require the involvement of upper or lower limb function. In this study, we only applied different interventions on the upper limbs between the two groups, but interventions for lower limb function were not restricted. In addition, most patients enrolled in this study presented with moderate to severe motor impairment, which might limit their improvement in functional independence of ADL [46].

In this study, the proprioceptive program combined passive and active movements with visual feedback. We conducted passive bimanual repetitive flexion-extension in the upper limbs of patients in a mirror-symmetrical pattern. Passive movements of unilateral upper limb could elicit brain activity in bilateral sensorimotor areas [47]. There are neural connections in the bilateral somatosensory cortex, which allow sensory information from both hands to transfer between hemispheres [48]. The level of brain activity of the affected side was decreased due to the inhibitory imbalance after stroke. Some studies have suggested that using mirror-symmetric bilateral movements as a priming approach could enhance the recovery of upper limb function by rebalancing the excitability of cortical activity [49, 50]. In these studies, patients were instructed to actively flex and extend their unaffected wrist, with a customized device driving their affected wrist in a synchronized mirror-symmetric or parallel movement pattern [51, 52]. While robot-assisted training could provide systematic procedures and various feedback, they are often customized for single-joint and the high cost may limit its application to clinical therapy. Compared with robot-assisted training, sensory training with guidance from therapists might be more feasible and suitable for clinical practice. Kiper et al. [18] reported that patients with stroke had significant improvement in muscle force after bilateral flexion-extension movements training mobilized by the therapist.

In addition to passive movement, we also applied active movement in our training procedure, which increased the challenge of training and patient engagement. Compared with passive proprioceptive training, active proprioceptive training with visual feedback allowed participants to update the internal model of the current state by constantly performing error detection and correction [25]. The common principles of active somatosensory interventions in previous studies were high intensity, repetitive, and attentive discrimination on the graded difficulty of sensory tasks with feedback [10]. Eye masks and verbal instructions were regularly used to provide feedback during training. However, frequent wearing and taking off the eye mask may make the patient lose patience and influence their sense of immersion and engagement. Some patients in the CG group also complained that closing their eyes for a long time made them feel tired and distracted. To overcome these limitations, we developed a visual-based sensory training setup that enabled therapists to switch the vision of patients instantly. The mode of the smart window can be converted in one second, which is convenient for therapists and patients. Patients were trained to actively discriminate different positions or movements of the affected limb when their vision was blocked and then receive visual feedback for self-checking. Moreover, patients were instructed to observe and sense the stimulation on their hands when the visual-based sensory training setup was in transparent mode. Previous studies have demonstrated that looking at the stimulated body part might induce short-term activation in somatosensory cortices [53]. With the help of immediate enhancement, patients could still sense proprioceptive stimuli without visual input for a while. Repeating the process might facilitate neural plasticity by enhancing experience-dependent synaptic connections. In our study, the visual-based sensory training setup provided a perceptual context in which the patient received visuo-proprioceptive stimulation and feedback. According to the result of “intention to use” in the modified meCUE, most patients were willing to use this setup for daily training.

Since patients had rarely received sensory training before, they were attracted by the setup. As indicated by the modified meCUE results, patients had positive product perceptions of usefulness, usability, and visual aesthetics. However, some patients indicated that the setup could be improved by increasing the space of the training platform and the size of the smart window. In our study, the setup only allowed patients to place their hands, wrists, and part of the forearms on the training platform, which limited the training on the rest of the upper limb. Future studies should consider developing a moveable smart window to make the setup more flexible.

There are some limitations to our study. First, the sample size was still small in this study, which affects the generalizability of the results. Second, the method of application of the setup and the training tasks was different between the two groups. There is no conclusive enough evidence that the visual feedback have some effect on the proprioceptive training in the present study. We could only provide preliminary results for it. In addition, patients were not blind to the assignment group, which might result in a placebo effect in this study.

In conclusion, we investigated the effectiveness and feasibility of the proposed proprioceptive training using visual-based sensory training setup in patients with poststroke. We found that proprioceptive training effectively improved the upper extremity sensorimotor function in stroke patients.

## Figures and Tables

**Table 1:** Demographics and characteristics at baseline of the patients with poststroke.

Characteristics	Total group (*N* = 40)	PG (*N* = 20)	CG (*N* = 20)	*p* value
Age (years)				
Mean ± SD	59.1 ± 11.4	58.4 ± 10.5	59.8 ± 12.5	0.704

Gender (*n*)				0.744
Male	25	13	12	
Female	15	7	8	

Time form stroke (weeks)				
Median (IQRs)	21 (31)	19 (30)	22 (33)	0.779

Side of lesion (*n*)				1.000
Left	16	8	8	
Right	24	12	12	

Type of stroke (*n*)				0.113
Hemorrhagic	19	12	7	
Ischemic	21	8	13	

Brunnstrom—upper extremity				
Median (IQRs)	3 (2)	3 (1)	2.5 (2)	0.947

Brunnstrom—hand				
Median (IQRs)	2 (3)	2 (3)	2 (3)	0.989

Somatosensory impairment (%)				
TLT	90	90	90	1.000
FMA-UE-S	85	90	80	0.658

**Table 2:** Modified meCUE.

Modules	Patients rating (*N* = 20) (mean ± SD)
Product perception	
Usefulness	5.12 ± 0.82
Usability	5.32 ± 1.28
Visual aesthetics	5.44 ± 0.93
Emotions	
Positive emotions	4.24 ± 0.88
Negative emotions	2.61 ± 1.13
Consequences of use	
Intention to use	4.95 ± 1.35
Overall evaluation	2.53 ± 1.57

## Data Availability

The data used to support the findings of this research are available from the corresponding author.
